# Integrated Chinese and Western medicine for stable angina pectoris of coronary heart disease: a real-world study including 690 patients

**DOI:** 10.3389/fcvm.2023.1194082

**Published:** 2023-05-19

**Authors:** Linghua Yu, Zihan Wang, Chenxi Xu, Anxiang Liu, Tong Li, Yubi Wang, Xiaoyan Lu, Hao Xu

**Affiliations:** ^1^ Graduate School, Beijing University of Chinese Medicine, Beijing, China; ^2^ Department of Integrative Cardiology, China-Japan Friendship Hospital, Beijing, China; ^3^ Xiyuan Hospital, China Academy of Chinese Medical Sciences, Beijing, China; ^4^ National Clinical Research Center for Chinese Medicine Cardiology, Xiyuan Hospital, China Academy of Chinese Medical Sciences, Beijing, China

**Keywords:** coronary heart disease, stable angina pectoris, cardiovascular risk, integrated treatment, logistic models, propensity score matching

## Abstract

**Objective:**

We aimed to evaluate the effects of integrated Chinese and Western medical therapeutic modalities on clinical prognosis in a population with stable angina pectoris (SAP) of coronary heart disease (CHD).

**Methods:**

In a prospective cohort study, 732 patients with SAP of CHD hospitalized in the Integrated Cardiology Unit of the China-Japan Friendship Hospital From October 2020 to October 2021 were included. The patients were divided into integrated treatment and conventional treatment groups according to whether they had been taking Chinese medicine for more than 6 months per year. The occurrence of composite cardiovascular events (CVEs), including cardiac death, non-fatal myocardial infarction, revascularization, stroke, all-cause death, and readmission due to angina attack, heart failure, or malignant arrhythmia, was recorded during follow-up. The effects of different treatment modalities on prognosis were evaluated using univariate and multifactorial logistic regression. Logistic regression models were evaluated using receiver operating characteristic (ROC) curves. In sensitivity analysis, the correlation between treatment modality and outcome events was corrected by rematching the two groups of patients using the propensity score matching (PSM) method.

**Results:**

The data from 690 patients were included in the analysis, with 327 patients in the integrated treatment group and 363 patients in the conventional treatment group. CVEs occurred in 19 patients (5.8%) in the integrated treatment group and 37 patients (10.2%) in the conventional treatment group. The proportion of outcome events was significantly lower in the combination treatment group than in the conventional treatment group (*P* = 0.037). Covariate correction by multimodal multifactorial logistic regression revealed a lower risk of CVEs in patients receiving integrated therapy compared with conventional therapy (OR = 0.246, 95% CI = 0.097–0.622, *P* = 0.003). Moreover, a history of renal insufficiency (OR = 3.991, 95% CI = 1.164–13.684, *P* = 0.028) and a higher Gensini score (OR = 1.039, 95% CI = 1.028–1.050, *P* < 0.001) were risk factors for the development of CVEs. Model evaluation showed that C-statistic = 0.955 and area under the ROC curve (AUC) = 0.955. After PSM correction, the results still showed that integrated Chinese and Western medical treatment reduced the occurrence of CVEs in patients compared with Western treatment alone (OR = 0.339, 95% CI = 0.131–0.874, *P* = 0.025).

**Conclusion:**

Integrated treatment based on Chinese and Western medicine might improve the prognosis and reduce the risk of CVEs in this disease population.

**Trial registration:**

China Clinical Trials Registry, ChiCTR1800017891, Registered 20 August 2018, http://www.chictr.org.cn/showproj.aspx?proj = 30170.

## 1. Introduction

In recent years, the prevalence of stable angina pectoris (SAP) of coronary heart disease (CHD) has been increasing in response to lifestyle changes and the progression of an aging population (1). SAP of CHD is defined as an acute, transient ischemic–hypoxic syndrome of the myocardium due to increased myocardial load on top of a fixed severe stenosis of the coronary arteries (2). The prognosis of patients with CHD alone or with SAP has been of great concern, and some studies (3) have shown that people with SAP of CHD are more severely ill and have a significantly increased risk of cardiovascular death and rehospitalization compared with people with CHD alone. Currently, for people with SAP of CHD, conventional treatment is aimed at symptomatic relief, with long-term use of oral antiplatelet, plaque-stabilizing, and vasodilatory drugs recommended, and severe patients requiring vascular intervention (4). However, data from large sample trials suggest that pharmacological treatment or coronary revascularization, while relieving clinical symptoms, is not effective in reducing the incidence of angina pectoris and composite cardiovascular events (CVEs) (5). Integrated Chinese and Western medicine has been found to be more effective in patients with complex conditions and has been shown to improve the prognosis of patients with SAP of CHD. Currently, high-quality randomized controlled trials are the main focus of investigations on the effectiveness and safety evaluation of traditional chinese medicine (TCM) therapies for SAP of CHD. Due to variations between the stringent treatments used in the trial and the actual patient settings, Randomized Controlled Trial (RCT) studies exhibit low repeatability of outcomes. It also makes sense to include multidimensional analysis metrics in the trial—simulating actual patient settings—to evaluate the prognosis of stable angina in coronary artery disease because the prevalence of CVEs is relative to various parameters. In a real-world study, patient settings could be shown objectively and realistically and the effectiveness and safety of TCM in complex clinical practice could be better assessed.

The aim of this study was to further investigate the possibilities to improve the prognosis of the population with SAP of CHD. We adopted a prospective cohort study to assess the incidence of CVEs in real-world patients with SAP of CHD, treated with integrated Chinese and Western medicine or conventional Western medicine, and examine whether there are differences in the risk of CVEs.

## 2. Materials and methods

### 2.1. Selection methods

This study is a prospective cohort study and was conducted following the guidelines laid down in the TRIPOD statement (6). A total of 827 patients with SAP of CHD hospitalized in the Integrated Cardiology Unit of the China-Japan Friendship Hospital from October 2020 to October 2021 were recruited. Exclusion criteria were as follows: data collection was affected by mental or language factors; heart failure; the presence of severe systemic diseases such as tumors; or expected survival time < 12 months. In total, 104 patients were excluded, so 723 patients were included. The inclusion process is shown in Supplementary Figure S1.

This study was approved by the Clinical Research Ethics Committee of the China-Japan Friendship Hospital (2020-114-K73), conducted in accordance with the Declaration of Helsinki, and registered with the China Clinical Trials Registry (ChiCTR1800017891). All patients signed informed consent and gave permission to use the data for further clinical studies.

### 2.2. Diagnostic criteria

Patients were diagnosed in reference to the “1979 Nomenclature and Diagnostic Criteria for Ischemic Heart Disease” (7) jointly issued by the International Society of Cardiology and the World Health Organization and the “2012 Guidelines for the Diagnosis and Treatment of Stable Ischemic Heart Disease” (8) jointly issued by the American Heart Association (AHA) and several other societies.

### 2.3. Research methods

#### 2.3.1. Basic information

General information was collected, including demographics (gender, age, height, weight), other relevant medical history (hypertensive disease, diabetes, hyperlipidemia, atherosclerosis of the carotid artery, stroke, renal insufficiency), and personal habits (smoking, alcohol consumption, daily physical activity). Physical activity was reported by the patients. High physical activity was defined as moderate-intensity physical activity for more than 60 min per day.

The 19-item Seattle Angina Questionnaire (SAQ) (9) was used to assess patients' quality of life. Information was collected from patients by an independent investigator. Five dimensions were assessed according to published criteria on an article-by-article basis: the degree of limitation of exertional capacity, anginal stability, anginal frequency, disease perception, and treatment satisfaction.

#### 2.3.2. Testing and examination

Venous blood specimens were taken from all patients on an empty stomach in the early morning of the second day after admission, and homocysteine (Hcy), low-density lipoprotein–cholesterol (LDL-C), lipoprotein a (LP-a), glycosylated hemoglobin (HbA1c), blood urea nitrogen, and serum creatinine (Scr) were tested at the Laboratory Department of the China-Japan Friendship Hospital. The last coronary angiography results were retrieved from the Medical Record System of the China-Japan Friendship Hospital to record the lesions of the vessels. The Gensini scores were calculated according to the Guidelines for Calculating Gensini Scores (10). All patients underwent coronary angiography.

#### 2.3.3. Therapy and grouping

No medication interventions were made in this study, and the treatment regimens were based on the prescriptions issued by the patients' primary care physicians. Usage of antiplatelet agents (aspirin, clopidogrel), anticoagulants (warfarin, dabigatran, rivaroxaban), antimyocardial ischemic drugs (trimetazidine, nicorandil), vasodilators (nitrates), ACEI/ARB *β*-blockers, calcium antagonists (CCB), and lipid-lowering drugs (statins, ezetimibe) was recorded.

On the basis of the above, some patients received traditional Chinese medicine treatment according to their own will or their doctor's recommendation. Traditional Chinese medicine included Chinese herbal soup and proprietary Chinese medicine, which were individually prescribed by the attending physician. Patients who continued to take Chinese medicine for more than 6 months per year during follow-up were recorded as “combined therapy”; otherwise, they were recorded as “conventional therapy”. All patients maintained continuous and regular treatment, with specific medications adjusted at follow-up visits.

#### 2.3.4. Follow-up and outcomes

Follow-up was performed by an independent investigator, with outpatient or telephone follow-up visits every 6 months after patients were enrolled in the study to collect information over time until the end of the 12-month study period. Patients whose medication regimen changed significantly during the follow-up period (three or more changes in the Western medication regimen), patients who withdrew on their own, patients who could not be contacted, and patients who were unable to cooperate in completing the follow-up were excluded from further analysis.

CVEs were used as the primary endpoint events in this study, including cardiac death, non-fatal myocardial infarction, revascularization, stroke, all-cause death, and readmission due to angina attack, heart failure, or malignant arrhythmia.

#### 2.3.5. Adverse reaction (AR)

AR events were defined as any adverse medical events that occurred during the follow-up period, whether they were causally related to drug use. During the follow-up, a small number of patients presented difficulties in stool, fatigue, liver function impairment, gastrointestinal bleeding, skin purpura and other unexpected events, which were uniformly recorded as AR.

#### 2.3.6. Sample calculation

A total of 35 variables were included in the research as factors that could potentially influence prognosis. Ultimately, 723 patients with SAP of CHD were included, in accordance with the principle of approximately 10 patients per group for each variable in the regression analysis (11).

### 2.4. Data analysis

Statistical analysis and visualization were performed in R (V4.1.1). Normally distributed data are expressed as mean ± standard deviation, non-normally distributed data are expressed as median (interquartile range), and count data are expressed as percentages. When comparing the means of two sample groups, the independent samples *t*-test was used for normally distributed data, the rank sum test (Kruskal–Wallis H) was used for non-normally distributed data, and the chi-square test was used for count data.

Patients who completed the follow-up were selected for per-protocol analysis (PPS). Multiple interpolation was performed using the MICE package in R for variables with missing values <20%. Logistic regression analysis was used to observe whether different treatment modalities had an impact on the outcome events. Univariate and multifactorial logistic regression was used to evaluate whether different treatment modalities affected patient prognosis. Logistic regression models were evaluated based on consistency indices (C-statistic) and time-dependent receiver operating characteristic (ROC) curves. At last, sensitivity analyses were performed. The two groups (different treatment modalities) were rematched based on propensity score matching (PSM) to minimize bias in the observed values and to correct for confounding factors, followed by a corrective test for the association between treatment modality and outcome events. Considering the variables of age, sex, BMI, physical activity, smoking, alcohol consumption and others, the score was calculated using a logistic regression model. A 1:1 ratio matching between the two groups was performed to maximize the PSM based on the nearest neighbor method. *P* < 0.05 was considered statistically significant.

## 3. Results

### 3.1. Study population

We excluded 33 (4.6%) patients with incomplete follow-up data. Follow-up statistical analyses were performed in the final PPS with 690 patients for whom complete data were available.

The number of patients was 327 in the integrated treatment group and 363 in the conventional treatment group. Baseline characteristics are shown in Table 1. The combined treatment group had a higher percentage of alcohol intake (*P* < 0.001), a lower percentage of hyperlipidemia (*P* = 0.034) and lipid-lowering medication (*P* = 0.001), and lower Scr values (*P* = 0.005) compared with the conventional treatment group. This reflects the clinical reality of the treatment of patients with SAP of CHD, where clinicians tend to use surgical methods (percutaneous coronary intervention) first to solve the main problem in patients with more complex conditions and start the combined intervention with herbs only after clarifying the overall situation, to avoid possible complications and adverse effects. The incidence of AR during treatment was also lower in the integrated treatment group than in the conventional treatment group (*P* = 0.014).

**Table 1:** Description of the clinical characteristics of the study population.

Variables	Total (*N* = 690)	Combined treatment (*N* = 327)	Conventional treatment (*N* = 363)	*P* values^a^
Sex [male, *N*(%)]	438 (63.5)	203 (62.1)	235 (64.7)	0.477
Age (years)	65.1 ± 11.32	65.13 ± 11.28	65.07 ± 11.38	0.942
BMI (Kg*m-2)	25.57 ± 1.69	25.51 ± 1.72	25.63 ± 1.67	0.382
Physical activity [Hgih, *N*(%)]	297 (43.0)	151 (46.2)	146 (40.2)	0.124
Smoking [*N*(%)]	427 (61.9)	201 (61.5)	226 (62.3)	0.875
Alcohol consumption [*N*(%)]	330 (47.8)	196 (59.9)	134 (36.9)	**<0**.**001**
Hypertension [*N*(%)]	280 (40.6)	130 (39.8)	150 (41.3)	0.698
Diabetes [*N*(%)]	234 (33.9)	104 (31.8)	130 (35.8)	0.295
Hyperlipemia [*N*(%)]	222 (32.2)	92 (28.1)	130 (35.8)	**0**.**034**
Carotid atherosclerosis [*N*(%)]	245 (35.5)	127 (38.8)	118 (32.5)	0.094
Stroke [*N*(%)]	78 (11.3)	37 (11.3)	41 (11.3)	<0.999
Renal insufficiency [*N*(%)]	28 (4.1)	12 (3.7)	16 (4.4)	0.701
Antiplatelet [*N*(%)]	599 (86.8)	287 (87.8)	312 (86.0)	0.501
Antianginal [*N*(%)]	101 (14.6)	46 (14.1)	55 (15.2)	0.747
Nitrate ester [*N*(%)]	142 (20.6)	67 (20.5)	75 (20.7)	<0.999
ACEI/ARB [*N*(%)]	303 (43.9)	140 (42.8)	163 (44.9)	0.592
*β*-blockers [*N*(%)]	367 (53.2)	171 (52.3)	196 (54.0)	0.703
CCB [*N*(%)]	206 (29.9)	94 (28.7)	112 (30.9)	0.561
Anticoagulant [*N*(%)]	62 (9.0)	25 (7.6)	37 (10.2)	0.286
Lipid-lowering [*N*(%)]	611 (88.6)	275 (84.1)	336 (92.6)	**0**.**001**
Gensini score	23.00 (10.00, 47.25)	23.00 (12.00, 50.00)	22.00 (10.00, 47.00)	0.471
Hcy (*μ*mol*L-1)	15.00 ± 7.39	15.16 ± 8.86	14.85 ± 5.77	0.584
LDL-C (mmol*L-1)	2.35 ± 0.71	2.38 ± 0.72	2.32 ± 0.70	0.247
Lp-a (mg*L-1)	124.02 (56.12, 223.89)	116.7 (56.12, 183.29)	133.77 (52.00, 253.86)	0.057
HbA1c (%)	6.34 ± 2.93	6.12 ± 1.31	6.53 ± 3.84	0.063
Urea (mmol*L-1)	17.36 ± 8.10	16.75 ± 8.31	17.91 ± 7.87	0.059
Scr (mg*dL-1)	0.96 (0.82, 1.15)	0.92 (0.81, 1.11)	0.99 (0.82, 1.17)	**0**.**005**
SAQ of Exertional capacity	61.67 ± 15.37	59.72 ± 16.81	61.32 ± 15.09	0.189
SAQ of Anginal stability	50.67 ± 22.13	52.25 ± 23.39	49.72 ± 21.19	0.755
SAQ of Anginal frequency	75.86 ± 20.76	75.50 ± 25.12	74.72 ± 16.24	0.653
SAQ of Disease perception	65.48 ± 17.13	64.13 ± 18.95	65.31 ± 15.31	0.367
SAQ of Treatment satisfaction	80.49 ± 11.37	80.13 ± 11.88	79.38 ± 11.43	0.399
AR [*N*(%)]	60 (8.7)	19 (5.8)	41 (11.3)	**0**.**014**
CVEs [*N*(%)]	56 (8.1)	19 (5.8)	37 (10.2)	**0**.**037**

^a^ Differential analysis of the data between the “Combined treatment” and “Conventional treatment” groups, with bolded values representing statistical significance.

### 3.2. Univariate and multivariate logistic regression analysis

At the end of the 12-month follow-up period, 56 (8.1%) CVEs were reported, with 19 (5.8%) CVEs in the combination treatment group and 37 (10.2%) CVEs in the conventional treatment group. The proportion of outcome events was significantly lower in the combination treatment group than in the conventional treatment group (*P* = 0.037).

We used univariate logistic regression to analyze the relevance of each variable to the outcome event (Supplementary Table S1). Furthermore, to eliminate confounding factors, we performed a multivariate logistic regression test based on the possible correlation between treatment modality and CVEs in multiple models. The variables with significance <0.2 in the univariate regression were regarded as potentially influential factors and were included as covariates in the model. Model I was corrected for gender; Model II was additionally corrected for physical activity, history of alcohol consumption, comorbidities (diabetes, renal insufficiency), underlying medication (antiplatelet, ACEI/ARBs, *β*-blockers) and AR; Model III was further corrected for the continuous variables of examination results (Gensini score, HbA1c, urea) and SAQ scores. Model III was used as the main study model. The results of the logistic regression tests are shown in Table 2.

**Table 2:** Logistic regression with multiple factors for multiple models.

Model	Variables	Beta	OR (95% CI)	*P* values
Model I	Sex			
	Male	Reference		
	Female	−0.791	0.453 (0.234–0.877)	0.019
	Combined treatment^a^	−0.596	0.551 (0.309–0.981)	0.043
Model II	Physical activity			
	Low	Reference		
	High	−0.912	0.402 (0.205–0.787)	0.008
	Antiplatelet			
	No	Reference		
	Yes	−2.272	0.103 (0.057–0.187)	<0.001
	Combined treatment^a^	−0.557	0.573 (0.310–1.059)	0.076
Model III	Physical activity			
	Low	Reference		
	High	−1.195	0.303 (0.118–0.775)	0.013
	Renal insufficiency			
	No	Reference		
	Yes	1.384	3.991 (1.164–13.684)	0.028
	Antiplatelet			
	No	Reference		
	Yes	−2.213	0.109 (0.044–0.272)	<0.01
	Gensini score	0.038	1.039 (1.028–1.050)	<0.01
	SAQ of Exertional capacity	−0.042	0.959 (0.933–0.986)	0.003
	SAQ of Treatment satisfaction	−0.072	0.931 (0.898–0.965)	<0.01
	Combined treatment^a^	−1.403	0.246 (0.097–0.622)	0.003

^a^ With conventional treatment as a reference.

After multivariate correction in Model III, we found that treatment modality was an independent factor of CVEs in patients with SAP with CHD (*P* < 0.001) and that combination therapy reduced the risk of CVEs compared with conventional therapy (OR = 0.246, 95% CI = 0.097–0.622, *P* = 0.003). In addition, physical activity (OR = 0.303, 95% CI = 0.118–0.775, *P* = 0.013) and regular use of antiplatelet medication (OR = 0.109, 95% CI = 0.044–0.272, *P* < 0.001) reduced the risk of CVEs, whereas a history of renal insufficiency (OR = 3.991, 95% CI = 1.164–13.684, *P* = 0.028) and a higher Gensini score (OR = 1.039, 95% CI = 1.028–1.050, *P* < 0.001) were risk factors for the development of CVEs. Higher Exertional capacity scores (OR = 0.959, 95% CI = 0.933–0.986, *P* = 0.003) and Treatment satisfaction scores (OR = 0.931, 95% CI = 0.898–0.965, *P* < 0.001) on SAQ were associated with a lower risk of CVEs.

It is the goal of TCM to increase efficacy and reduce side effects, so we paid extra attention to the occurrence of AR during the follow-up period, and built a regression model in the similar way to determine whether different treatment methods are related to AR. The results showed that combined therapy could reduce the risk of AR (Supplementary Table S2, OR = 0.508, 95% CI = 0.283–0.910, *P* = 0.023). Meanwhile, there was no significant change in the incidence of CVEs in patients with or without AR (CVEs: 1.7% vs. 8.7%, *P* = 0.078).

In a more detailed analysis of the outcome events, we found that angina attack (*N* = 36, 64.3%) had the highest proportion of CVEs, followed by all-cause death (*N* = 9, 16.1%). Therefore, we analyzed the outcomes separately, as a secondary focus. We found no significant difference in the type of outcome between treatments (angina attack: 63.2% vs. 64.9%, *P *> 0.999; all-cause death: 16.2% vs. 15.8%, *P *> 0.999). Different treatment methods, renal insufficiency, antiplatelet, Gensini score, Exertional capacity scores and Treatment satisfaction scores are the influencing factors (Supplementary Table S3) for angina attack. The remaining separative outcomes could not be analyzed effectively due to the small number of cases.

### 3.3. Model evaluation

**Figure 1:**
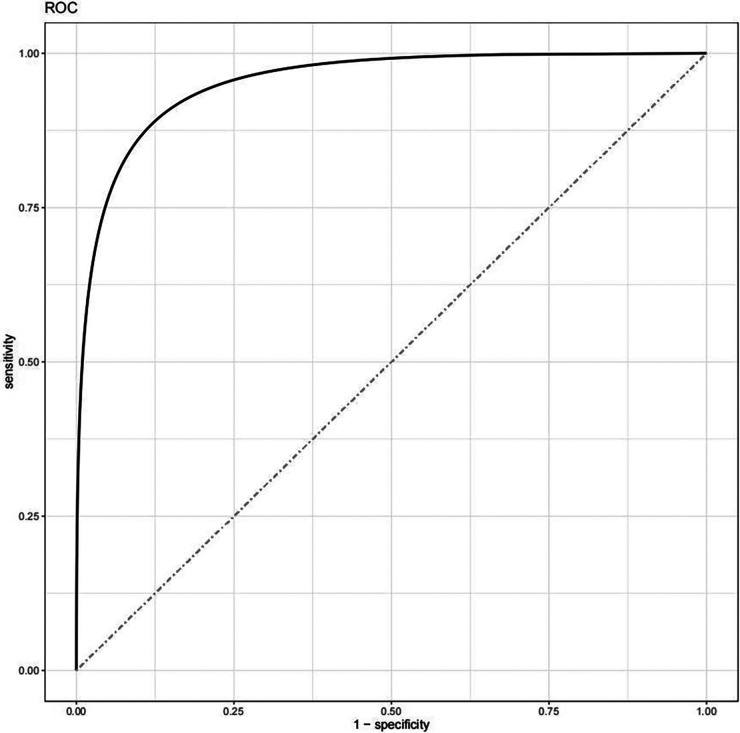
The ROC curve is used to evaluate the discrimination of the model. Full line: the logistic regression model (Model III), AUC = 0.955, Dotted line: Reference line.

The logistic regression model (Model III) was statistically evaluated with a negative 2-fold log-likelihood value, and the Omnibus test showed that *χ*^2^ = 212.72 (*P* < 0.001), indicating that the overall result of the model was statistically significant. The discrimination of the model was tested and we found that C-statistic = 0.955. We used ROC curves based on the proportion of CVEs during follow-up (Figure 1) for model evaluation; the fitted ROC curves gave similar results to the C-statistic, with an area under the curve (AUC) of 0.955. These results demonstrate that Model III is highly accurate and can predict the occurrence of CVEs in patients.

### 3.4. Sensitivity analysis

Real-world data may suffer from baseline bias. We observed differences in alcohol consumption, hyperlipemia, usage of lipid-lowering drugs, and Scr between the two groups (Table 1). We performed PSM in PPS to minimize bias in the observations and reduce the influence of confounding factors. The baseline profiles of the two groups of patients after matching are shown in Table 3.

**Table 3:** Clinical characterization of the study population in PSM.

Variables	Total (*N* = 544)	Combined treatment (*N* = 272)	Conventional treatment (*N* = 272)	*P* values^a^
Sex [male, *N*(%)]	320 (58.8)	161 (59.2)	159 (49.7)	0.862
Age (years)	65.35 ± 11.13	65.31 ± 11.24	65.42 ± 11.04	0.905
BMI (Kg*m-2)	25.53 ± 1.71	25.55 ± 1.78	25.51 ± 1.65	0.799
Physical activity [Hgih, *N*(%)]	236 (43.4)	120 (44.1)	116 (42.6)	0.729
Smoking [*N*(%)]	348 (64.0)	173 (63.6)	175 (64.3)	0.858
Alcohol consumption [*N*(%)]	271 (49.8)	144 (52.9)	127 (46.7)	0.145
Hypertension [*N*(%)]	213 (39.2)	104 (38.2)	109 (40.1)	0.661
Diabetes [*N*(%)]	185 (34.0)	90 (33.1)	95 (34.9)	0.651
Hyperlipemia [*N*(%)]	177 (32.5)	83 (30.5)	94 (34.6)	0.314
Carotid atherosclerosis [*N*(%)]	177 (32.5)	93 (34.2)	84 (30.9)	0.410
Stroke [*N*(%)]	59 (10.8)	29 (10.7)	30 (11.0)	0.890
Renal insufficiency [*N*(%)]	18 (3.3)	9 (3.3)	9 (3.3)	>0.999
Antiplatelet [*N*(%)]	475 (87.3)	240 (88.2)	235 (86.4)	0.519
Antianginal [*N*(%)]	85 (15.6)	39 (14.3)	46 (16.9)	0.408
Nitrate ester [*N*(%)]	117 (21.5)	54 (19.9)	63 (23.2)	0.348
ACEI/ARB [*N*(%)]	239 (43.9)	117 (43.0)	122 (44.9)	0.666
β-blockers [*N*(%)]	279 (51.3)	143 (52.6)	136 (50.0)	0.548
CCB [*N*(%)]	153 (28.1)	77 (28.3)	76 (27.9)	0.924
Anticoagulant [*N*(%)]	43 (7.9)	23 (8.5)	20 (7.4)	0.634
Lipid-lowering [*N*(%)]	493 (90.6)	247 (90.8)	246 (90.4)	0.883
Gensini score	22.00 (10.00, 47.00)	23.00 (12.00, 50.00)	22.00 (10.00, 45.50)	0.509
Hcy (μmol*L-1)	14.77 ± 6.60	14.68 ± 7.20	14.87 ± 5.95	0.731
LDL-C (mmol*L-1)	2.37 ± 0.72	2.36 ± 0.73	2.38 ± 0.71	0.710
Lp-a (mg*L-1)	119.57 (56.12, 222.66)	115.88 (56.12, 195.15)	129.85 (53.07, 235.56)	0.253
HbA1c (%)	6.27 ± 2.19	6.17 ± 1.37	6.36 ± 2.78	0.320
Urea (mmol*L-1)	17.32 ± 7.65	17.20 ± 8.84	17.44 ± 6.26	0.716
Scr (mg*dL-1)	0.96 (0.81, 1.11)	0.92 (0.81, 1.09)	0.98 (0.80, 1.12)	0.075
SAQ of Exertional capacity	60.17 ± 15.96	60.67 ± 16.22	59.67 ± 15.70	0.467
SAQ of Anginal stability	50.36 ± 22.49	50.80 ± 23.47	49.92 ± 21.50	0.646
SAQ of Anginal frequency	74.63 ± 22.15	75.50 ± 26.51	73.76 ± 16.69	0.360
SAQ of Disease perception	64.69 ± 17.17	65.03 ± 18.55	64.35 ± 15.70	0.644
SAQ of Treatment satisfaction	79.93 ± 11.71	79.91 ± 11.80	79.96 ± 11.64	0.963
CVE [*N*(%)]	42 (7.7)	16 (5.9)	26 (9.6)	0.108

^a^ Differential analysis of the data between the “Combined treatment” and “Conventional treatment” groups.

Analysis process of the data in Section 3.2 was repeated using the post-PSM dataset. Variables with significance <0.2 in the univariate regression (Supplementary Table S4) were regarded as potential influences and directly included in the multivariate regression model as covariates. The final results are shown in Table 4. After PSM processing, no difference was found in outcome events (CVEs: 5.9% vs. 9.6%, *P *= 0.108). We considered that the differences between different groups were masked by confounding factors in the single-factor Chi-square test. The results of the sensitivity analysis (regression methods) were similar to those produced by the main model (Model III), showing a statistically significant impact of treatment modality on the occurrence of CVEs, with the combination of Chinese and Western medicine acting as a protective factor against CVEs (OR = 0.339, 95% CI = 0.131–0.874, *P* = 0.025).

**Table 4:** Logistic regression with multiple factors in PSM.

Variables	Beta	OR (95% CI)	*P* values
Physical activity			
Low	Reference		
High	−1.189	0.305 (0.110–0.844)	0.022
Antiplatelet			
No	Reference		
Yes	−1.849	0.157 (0.058–0.423)	<0.001
Gensini score	0.037	1.038 (1.026–1.050)	<0.001
SAQ of Exertional capacity	−0.046	0.955 (0.927–0.984)	0.002
SAQ of Treatment satisfaction	−0.077	0.926 (0.891–0.961)	<0.001
Combined treatment^a^	−1.083	0.339 (0.131–0.874)	0.025

^a^ With conventional treatment as a reference.

## 4. Discussion

The effectiveness of TCM in the treatment of cardiovascular disease has been widely demonstrated in clinical practice (12–16). Despite high-quality randomized controlled trials being used in the current research, enhancing the external validity of clinical research and minimizing the differences between the trial and the real world are still necessary for translating research findings into clinical practice. In order to lessen the disparity between trial conditions and complex clinical practice, this research adopted a prospective cohort study based on the real world to record and collect clinical data from patients with SAP in detail, and cross-checked by single-factor and multi-factor logistic regression analysis for a more objective evaluation of the effectiveness and safety of integrated Chinese and Western medicine in the treatment of SAP.

Clinical characteristics showed that elderly men formed the majority of patients included in this study, most of whom had a combination of hypertension, diabetes mellitus, carotid atherosclerosis, and hyperlipidemia, with a few sufferings from stroke and renal insufficiency, suggesting the complexity of the general profile of the population in patients with SAP of CHD. Moreover, we observed that most patients' lifestyles could be improved, in terms of insufficient daily physical activity, consumption of tobacco and alcohol, etc. In particular, smoking, alcohol consumption, and excessive obesity are serious risk factors for cardiovascular disease, accelerating disease progression and increasing the risk of death (17–19). This suggests that it is necessary not only to improve treatment but also to guide patients to improve their lifestyles.

In the baseline information of the two groups, a higher proportion of alcohol consumption (*P* < 0.001), a lower proportion of hyperlipidemia (*P* = 0.034) and lipid-lowering medication use (*P* = 0.001), and lower Scr values (*P* = 0.005) were observed in the integrated treatment group than in the conventional treatment group. It was considered that the uneven distribution of covariates between the two groups occurred due to the non-randomized design used in this study. However, the results also reflect the current treatment principles of integrated Chinese and Western medicine therapies. For patients with more complicated conditions, to reduce adverse effects of integrated medications, clinicians give priority to standardized treatment protocols to solve the main problem and give Chinese herbs after clarifying the overall condition of the patient.

In this study, composite CVEs were selected as an outcome indicator (20), and we explored whether the integrated of Chinese and Western medicine is an independent influencing factor for the occurrence of composite endpoint events in patients with SAP of CHD. To reduce the confounding effects may existing among the variables, the variables obtained from the screening were further gradually included in several multivariate logistic regression models for testing. It was finally concluded that treatment modality was an independent factor for CVEs (*P* < 0.001). We also revealed that physical activity, regular use of antiplatelet drugs, history of renal insufficiency, a higher Gensini score, and some SAQ items were independently related to disease prognosis. Available studies (21, 22) have proved the role of regular moderate- to high-intensity exercise in the recovery of patients with cardiovascular disease. Antiplatelet agents are the basis of secondary prevention programs for patients with CHD and may improve disease prognosis (23). Renal function is an important concern for all patients with cardiovascular disease (24), and the two are closely related through the renin–angiotensin system, as explained in Chinese medicine as “Intercourse Between Heart and Kidney” (25–27) and “Heart and Kidney in Balance,” requiring attention in clinical practice. The Gensini score is the golden standard to evaluate the degree of coronary vascular disease (28), and patients with indications for coronary intervention are advised to undergo coronary intervention, and it has been proven to be effective in predicting the incidence of CVEs (29). SAQ scoring is one of the characteristic assessment methods, with the advantages of non-invasiveness and comprehensiveness; nevertheless, the SAQ score is not commonly used (30, 31). Our statistically significant results suggest the necessity for promoting this type of evaluation in the future in assessing the clinical status of patients with SAP of CHD.

In clinical trials, the interventions, such as innovative operations and complex combined interventions, are often specific and cannot be randomized. PSM (32) is commonly introduced to obtain the “net effect” between two dependent variables for near randomization of covariates and solving the uneven distribution of variables between two groups. To address the uneven distribution of some baseline data between the two cohorts in this study (proportion of alcohol consumption, proportion of hyperlipidemia, use of lipid-lowering medication, Scr), PSM was used in sensitivity analysis for baseline data processing. Notably, no statistical significance in terms of outcome events was observed in the two groups after matching. We considered this as a result of the effect of treatment modality on outcome events being masked by certain confounding factors in the baseline data (such as Gensini score and SAQ score) during the univariate analysis of variance. Given this, multifactor logistic regression tests were repeated for the matched dataset to further reduce the influence of confounding factors. We confirmed that therapeutic modality is indeed statistically relevant to the occurrence of CVEs.

Our real-world prospective cohort study adequately preserved the individualized treatment characteristics in an objective and realistic clinical treatment environment. The relatively broad inclusion and exclusion criteria may increase the heterogeneity of the included population. In addition, the group of combined therapy is integrated with individualized Chinese medicine treatment on the basis of conventional Western medicine treatment, which is under the guidance of Syndrome differentiation and treatment. To avoid irregularities in medication administration, each patient in the combined treatment group was prescribed Chinese herbal medicines by the doctor in charge, during which the prescription was adjusted according to the changes in the patient's symptoms. This also reflects the individualized and dynamic evolution of TCM in real-world studies. To control the influence of confounding factors on the results due to the non-randomized trial design, we applied PSM. In addition, physical activity, the Gensini score, the SAQ score, and other observations were included, as these indicators can objectively reflect the disease status of patients with SAP of CHD and are closely related to the prognosis of cardiovascular disease (33–35).

Nonetheless, this study has limitations. The follow-up period of this trial was short and only reflected the clinical effectiveness and safety of drug administration in subjects over a 1-year period. In addition, the single-center design of the trial allowed for a limited sample size and insufficient external validity of the model. In addition, the problem of heterogeneity in the combined drug group cannot be solved yet, which is one of the objective limitations of TCM in real-world studies. In the future, a multicenter, large-sample real-world research will be required to enhance the external validity of our model. Meanwhile, the follow-up period should be appropriately prolonged to further validate the effectiveness of integrated Chinese and Western medicine on the long-term prognosis of patients with SAP of CHD. Additionally, we will record each patient's prescription for medication in detail in future studies and consider setting up subgroup analyses based on the specific use of herbal medicines by patients to improve the credibility of the article.

## 5. Conclusion

We investigated the effect of real-world combined Chinese and Western medical therapy vs. conventional therapy on patient outcome events in patients with stable angina pectoris. We also developed multifactor logistic regression tests to explore the risk factors for the occurrence of stable angina pectoris in patients. Ultimately, we conclude that treatment modality is an independent factor influencing the occurrence of CVEs in patients with stable angina pectoris of coronary artery disease. Integrated treatment based on Chinese and Western medicine might improve the prognosis and reduce the risk of CVEs in this disease population.

## Data Availability

The original contributions presented in the study are included in the article/**Supplementary Material**, further inquiries can be directed to the corresponding author/s.
