# Supplementary material for: Integrated Chinese and Western medicine for stable angina pectoris of coronary heart disease: a real-world study including 690 patients
Source: Front Cardiovasc Med. 2023 May 19;10:1194082. doi: 10.3389/fcvm.2023.1194082 (PMC10235782; doi:10.3389/fcvm.2023.1194082)
Supplement: Supplementary file 1 [file Table1.docx]

***Supplementary Material***

**Integrated Chinese and Western medicine for stable angina pectoris of coronary artery disease: a real-world study including 690 patients**

Linghua Yu^1†^, Zihan Wang^1†^, Meishan Lu^1^, Chenxi Xu^1^, Anxiang Liu^1^, Tong Li^1^, Yubi Wang^1,^ Xinyi Zhou^1^, Lin Li^2^, Xiaoyan Lu^2*^, Hao Xu^3,4*^

*^1^Graduate School, Beijing University of Chinese Medicine, Beijing, People’s Republic of China.*

*^2^Department of Integrative Cardiology, China-Japan Friendship Hospital, Beijing, People’s Republic of China.*

*^3^Xiyuan Hospital, China Academy of Chinese Medical Sciences, Beijing, People’s Republic of China.*

*^4^National Clinical Research Center for Chinese Medicine Cardiology，Beijing，People’s Republic of China.*

**Equal contribution and first authorship:** Linghua Yu and Zihan Wang contributed equally to this work and share first authorship.

**Corresponding author:**

*Xiaoyan Lu: Tel: 010-84205041, E-mail: deerxiaoyan@126.com

*Hao Xu: Tel: 010-62835303, E-mail: [xuhaotcm@hotmail.com](mailto:xuhaotcm@hotmail.com)

**Table S1.** **Logistic regression of univariate**

| Variables | Beta | OR (95% CI) | *P* values |
| --- | --- | --- | --- |
| Sex |  |  |  |
| Male | Reference |  |  |
| Female | -0.804 | 0.448 (0.232-0.865) | **0.017** |
| Age | -0.002 | 0.998 (0.974-1.023) | 0.886 |
| BMI | 0.015 | 1.015 (0.863-1.193) | 0.86 |
| Physical activity |  |  |  |
| Low | Reference |  |  |
| High | -0.987 | 0.373 (0.197-0.706) | **0.002** |
| Smoking |  |  |  |
| No | Reference |  |  |
| Yes | 0.198 | 1.218 (0.685-2.167) | 0.501 |
| Alcohol consumption |  |  |  |
| No | Reference |  |  |
| Yes | -0.379 | 0.685 (0.392-1.197) | **0.184** |
| Hypertension |  |  |  |
| No | Reference |  |  |
| Yes | -0.059 | 0.943 (0.539-1.650) | 0.837 |
| Diabetes |  |  |  |
| No | Reference |  |  |
| Yes | 0.495 | 1.640 (0.944-2.849) | **0.079** |
| Hyperlipemia |  |  |  |
| No | Reference |  |  |
| Yes | 0.172 | 1.188 (0.671-2.104) | 0.554 |
| Carotid atherosclerosis |  |  |  |
| No | Reference |  |  |
| Yes | -0.163 | 0.849 (0.474-1.523) | 0.583 |
| Stroke |  |  |  |
| No | Reference |  |  |
| Yes | -0.283 | 0.753 (0.291-1.949) | 0.559 |
| Renal insufficiency |  |  |  |
| No | Reference |  |  |
| Yes | 1.428 | 4.170 (1.689-10.293) | **0.002** |
| Combined treatment |  |  |  |
| No | Reference |  |  |
| Yes | -0.61 | 0.554 (0.306-0.966) | **0.038** |
| Antiplatelet |  |  |  |
| No | Reference |  |  |
| Yes | -2.293 | 0.101 (0.056-0.181) | **＜0.001** |
| Antianginal |  |  |  |
| No | Reference |  |  |
| Yes | 0.39 | 1.478 (0.737-2.963) | 0.272 |
| Nitrate ester |  |  |  |
| No | Reference |  |  |
| Yes | 0.056 | 1.057 (0.543-2.060) | 0.87 |
| ACEI/ARB |  |  |  |
| No | Reference |  |  |
| Yes | 0.58 | 1.786 (1.028-3.102) | **0.04** |
| β-blockers |  |  |  |
| No | Reference |  |  |
| Yes | -0.373 | 0.689 (0.397-1.193) | **0.183** |
| CCB |  |  |  |
| No | Reference |  |  |
| Yes | 0.026 | 1.026 (0.566-1.860) | 0.932 |
| Anticoagulant |  |  |  |
| No | Reference |  |  |
| Yes | -0.008 | 0.992 (0.381-2.587) | 0.988 |
| Lipid-lowering |  |  |  |
| No | Reference |  |  |
| Yes | 0.556 | 1.744 (0.613-4.960) | 0.297 |
| Gensini score | 0.041 | 1.041 (1.033-1.050) | **＜0.001** |
| Hcy | 0.005 | 1.005 (0.971-1.039) | 0.79 |
| LDL-C | 0.043 | 1.044 (0.714-1.526) | 0.826 |
| Lp-a | -0.001 | 0.999 (0.998-1.001) | 0.536 |
| HbA1c | 0.055 | 1.056 (0.998-1.118) | **0.057** |
| Urea | 0.027 | 1.028 (1.002-1.053) | **0.033** |
| Scr | 0.079 | 1.082 (0.905-1.293) | 0.386 |
| SAQ of Exertional capacity | -0.056 | 0.946 (0.930-0.962) | **＜0.001** |
| SAQ of Anginal stability | -0.019 | 0.981 (0.969-0.993) | **0.002** |
| SAQ of Anginal frequency | -0.027 | 0.973 (0.960-0.987) | **＜0.001** |
| SAQ of Disease perception | -0.012 | 0.988 (0.973-1.002) | **0.097** |
| SAQ of Treatment satisfaction | -0.063 | 0.939 (0.917-0.961) | **＜0.001** |

Bolded values represent P values < 0.2.

**Table S2.** **Clinical characterization of the study population in PSM**

| Variables | Total (N=544) | Combined treatment (N=272) | Conventional treatment (N=272) | P values^△^ |
| --- | --- | --- | --- | --- |
| Sex [male,N(%)] | 320 (58.8) | 161 (59.2) | 159 (49.7) | 0.862 |
| Age (years) | 65.35±11.13 | 65.31±11.24 | 65.42±11.04 | 0.905 |
| BMI (Kg*m-2) | 25.53±1.71 | 25.55±1.78 | 25.51±1.65 | 0.799 |
| Physical activity [Hgih, N(%)] | 236 (43.4) | 120 (44.1) | 116 (42.6) | 0.729 |
| Smoking [N(%)] | 348 (64.0) | 173 (63.6) | 175 (64.3) | 0.858 |
| Alcohol consumption [N(%)] | 271 (49.8) | 144 (52.9) | 127 (46.7) | 0.145 |
| Hypertension [N(%)] | 213 (39.2) | 104 (38.2) | 109 (40.1) | 0.661 |
| Diabetes [N(%)] | 185 (34.0) | 90 (33.1) | 95 (34.9) | 0.651 |
| Hyperlipemia [N(%)] | 177 (32.5) | 83 (30.5) | 94 (34.6) | 0.314 |
| Carotid atherosclerosis [N(%)] | 177 (32.5) | 93 (34.2) | 84 (30.9) | 0.41 |
| Stroke [N(%)] | 59 (10.8) | 29 (10.7) | 30 (11.0) | 0.89 |
| Renal insufficiency [N(%)] | 18 (3.3) | 9 (3.3) | 9 (3.3) | 1 |
| Antiplatelet [N(%)] | 475 (87.3) | 240 (88.2) | 235 (86.4) | 0.519 |
| Antianginal [N(%)] | 85 (15.6) | 39 (14.3) | 46 (16.9) | 0.408 |
| Nitrate ester [N(%)] | 117 (21.5) | 54 (19.9) | 63 (23.2) | 0.348 |
| ACEI/ARB [N(%)] | 239 (43.9) | 117 (43.0) | 122 (44.9) | 0.666 |
| β-blockers [N(%)] | 279 (51.3) | 143 (52.6) | 136 (50.0) | 0.548 |
| CCB [N(%)] | 153 (28.1) | 77 (28.3) | 76 (27.9) | 0.924 |
| Anticoagulant [N(%)] | 43 (7.9) | 23 (8.5) | 20 (7.4) | 0.634 |
| Lipid-lowering [N(%)] | 493 (90.6) | 247 (90.8) | 246 (90.4) | 0.883 |
| Gensini score | 22.00 (10.00, 47.00) | 23.00 (12.00, 50.00) | 22.00 (10.00, 45.50) | 0.509 |
| Hcy (μmol*L-1) | 14.77±6.60 | 14.68±7.20 | 14.87±5.95 | 0.731 |
| LDL-C (mmol*L-1) | 2.37±0.72 | 2.36±0.73 | 2.38±0.71 | 0.71 |
| Lp-a (mg*L-1) | 119.57 (56.12, 222.66) | 115.88 (56.12, 195.15) | 129.85 (53.07, 235.56) | 0.253 |
| HbA1c (%) | 6.27±2.19 | 6.17±1.37 | 6.36±2.78 | 0.32 |
| Urea (mmol*L-1) | 17.32±7.65 | 17.20±8.84 | 17.44±6.26 | 0.716 |
| Scr (mg*dL-1) | 0.96 (0.81, 1.11) | 0.92 (0.81, 1.09) | 0.98 (0.80, 1.12) | 0.075 |
| SAQ of Exertional capacity | 60.17±15.96 | 60.67±16.22 | 59.67±15.70 | 0.467 |
| SAQ of Anginal stability | 50.36±22.49 | 50.80±23.47 | 49.92±21.50 | 0.646 |
| SAQ of Anginal frequency | 74.63±22.15 | 75.50±26.51 | 73.76±16.69 | 0.36 |
| SAQ of Disease perception | 64.69±17.17 | 65.03±18.55 | 64.35±15.70 | 0.644 |
| SAQ of Treatment satisfaction | 79.93±11.71 | 79.91±11.80 | 79.96±11.64 | 0.963 |
| CVE [N(%)] | 42 (7.7) | 16 (5.9) | 26 (9.6) | 0.108 |

^△^Differential analysis of the data between the “Combined treatment” and “Conventional treatment” groups.

**Table S3. Logistic regression of univariate in PSM**

| Variables | Beta | OR (95% CI) | *P* values |
| --- | --- | --- | --- |
| Sex |  |  |  |
| Male | Reference |  |  |
| Female | -0.731 | 0.481 (0.237-0.980) | **0.044** |
| Age | 0.005 | 1.005 (0.976-1.034) | 0.753 |
| BMI | -0.036 | 0.964 (0.802-1.159) | 0.699 |
| Physical activity |  |  |  |
| Low | Reference |  |  |
| High | -0.963 | 0.382 (0.184-0.793) | **0.01** |
| Smoking |  |  |  |
| No | Reference |  |  |
| Yes | -0.127 | 0.880 (0.463-1.673) | 0.698 |
| Alcohol consumption |  |  |  |
| No | Reference |  |  |
| Yes | -0.272 | 0.762 (0.404-1.439) | 0.402 |
| Hypertension |  |  |  |
| No | Reference |  |  |
| Yes | -0.048 | 0.953 (0.498-1.821) | 0.884 |
| Diabetes |  |  |  |
| No | Reference |  |  |
| Yes | 0.514 | 1.672 (0.886-3.157) | 0.113 |
| Hyperlipemia |  |  |  |
| No | Reference |  |  |
| Yes | 0.265 | 1.303 (0.680-2.498) | 0.425 |
| Carotid atherosclerosis |  |  |  |
| No | Reference |  |  |
| Yes | -0.202 | 0.817 (0.408-1.637) | 0.569 |
| Stroke |  |  |  |
| No | Reference |  |  |
| Yes | 0.114 | 1.121 (0.423-2.974) | 0.818 |
| Renal insufficiency |  |  |  |
| No | Reference |  |  |
| Yes | 1.3 | 3.669 (1.151-11.695) | **0.028** |
| Combined treatment |  |  |  |
| No | Reference |  |  |
| Yes | -0.525 | 0.591 (0.310-1.129) | **0.111** |
| Antiplatelet |  |  |  |
| No | Reference |  |  |
| Yes | -2.011 | 0.134 (0.068-0.263) | **＜0.001** |
| Antianginal |  |  |  |
| No | Reference |  |  |
| Yes | 0.261 | 1.299 (0.579-2.912) | 0.526 |
| Nitrate ester |  |  |  |
| No | Reference |  |  |
| Yes | -0.164 | 0.848 (0.382-1.886) | 0.687 |
| ACEI/ARB |  |  |  |
| No | Reference |  |  |
| Yes | 0.577 | 1.780 (0.942-3.363) | **0.076** |
| β-blockers |  |  |  |
| No | Reference |  |  |
| Yes | -0.263 | 0.769 (0.409-1.447) | 0.415 |
| CCB |  |  |  |
| No | Reference |  |  |
| Yes | 0.024 | 1.024 (0.510-2.056) | 0.947 |
| Anticoagulant |  |  |  |
| No | Reference |  |  |
| Yes | 0.223 | 1.250 (0.424-3.683) | 0.686 |
| Lipid-lowering |  |  |  |
| No | Reference |  |  |
| Yes | 0.318 | 1.374 (0.409-4.616) | 0.607 |
| Gensini score | 0.04 | 1.041 (1.031-1.051) | **＜0.001** |
| Hcy | 0.015 | 1.015 (0.976-1.055) | 0.45 |
| LDL-C | -0.25 | 0.799 (0.486-1.249) | 0.3 |
| Lp-a | -0.001 | 0.999 (0.996-1.001) | 0.217 |
| HbA1c | 0.002 | 1.002 (0.870-1.153) | 0.981 |
| Urea | 0.022 | 1.022 (0.990-1.055) | **0.177** |
| Scr | -0.03 | 0.971 (0.673-1.400) | 0.874 |
|  |  |  |  |
| SAQ of Exertional capacity | -0.061 | 0.941 (0.921-0.960) | **＜0.001** |
| SAQ of Anginal stability | -0.02 | 0.980 (0.967-0.994) | **0.004** |
| SAQ of Anginal frequency | -0.029 | 0.972 (0.957-0.987) | **＜0.001** |
| SAQ of Disease perception | -0.016 | 0.984 (0.968-1.000) | **0.054** |
| SAQ of Treatment satisfaction | -0.074 | 0.929 (0.904-0.954) | **＜0.001** |

Bolded values represent *P* values < 0.2.
