# Supplementary material for: Integrated Chinese and Western medicine for stable angina pectoris of coronary heart disease: a real-world study including 690 patients
Source: Front Cardiovasc Med. 2023 May 19;10:1194082. doi: 10.3389/fcvm.2023.1194082 (PMC10235782; doi:10.3389/fcvm.2023.1194082)
Supplement: Supplementary file 2 [file Table2.docx]

**Supplementary materials**

**Table S1.** **Logistic regression of univariate**

| Variables | Beta | OR (95% CI) | *P* values |
| --- | --- | --- | --- |
| Sex |  |  |  |
| Male | Reference |  |  |
| Female | -0.804 | 0.448 (0.232-0.865) | **0.017** |
| Age | -0.002 | 0.998 (0.974-1.023) | 0.886 |
| BMI | 0.015 | 1.015 (0.863-1.193) | 0.860 |
| Physical activity |  |  |  |
| Low | Reference |  |  |
| High | -0.987 | 0.373 (0.197-0.706) | **0.002** |
| Smoking |  |  |  |
| No | Reference |  |  |
| Yes | 0.198 | 1.218 (0.685-2.167) | 0.501 |
| Alcohol consumption |  |  |  |
| No | Reference |  |  |
| Yes | -0.379 | 0.685 (0.392-1.197) | **0.184** |
| Hypertension |  |  |  |
| No | Reference |  |  |
| Yes | -0.059 | 0.943 (0.539-1.650) | 0.837 |
| Diabetes |  |  |  |
| No | Reference |  |  |
| Yes | 0.495 | 1.640 (0.944-2.849) | **0.079** |
| Hyperlipemia |  |  |  |
| No | Reference |  |  |
| Yes | 0.172 | 1.188 (0.671-2.104) | 0.554 |
| Carotid atherosclerosis |  |  |  |
| No | Reference |  |  |
| Yes | -0.163 | 0.849 (0.474-1.523) | 0.583 |
| Stroke |  |  |  |
| No | Reference |  |  |
| Yes | -0.283 | 0.753 (0.291-1.949) | 0.559 |
| Renal insufficiency |  |  |  |
| No | Reference |  |  |
| Yes | 1.428 | 4.170 (1.689-10.293) | **0.002** |
| Combined treatment |  |  |  |
| No | Reference |  |  |
| Yes | -0.610 | 0.554 (0.306-0.966) | **0.038** |
| Antiplatelet |  |  |  |
| No | Reference |  |  |
| Yes | -2.293 | 0.101 (0.056-0.181) | **＜0.001** |
| Antianginal |  |  |  |
| No | Reference |  |  |
| Yes | 0.390 | 1.478 (0.737-2.963) | 0.272 |
| Nitrate ester |  |  |  |
| No | Reference |  |  |
| Yes | 0.056 | 1.057 (0.543-2.060) | 0.870 |
| ACEI/ARB |  |  |  |
| No | Reference |  |  |
| Yes | 0.580 | 1.786 (1.028-3.102) | **0.040** |
| β-blockers |  |  |  |
| No | Reference |  |  |
| Yes | -0.373 | 0.689 (0.397-1.193) | **0.183** |
| CCB |  |  |  |
| No | Reference |  |  |
| Yes | 0.026 | 1.026 (0.566-1.860) | 0.932 |
| Anticoagulant |  |  |  |
| No | Reference |  |  |
| Yes | -0.008 | 0.992 (0.381-2.587) | 0.988 |
| Lipid-lowering |  |  |  |
| No | Reference |  |  |
| Yes | 0.556 | 1.744 (0.613-4.960) | 0.297 |
| Gensini score | 0.041 | 1.041 (1.033-1.050) | **＜0.001** |
| Hcy | 0.005 | 1.005 (0.971-1.039) | 0.790 |
| LDL-C | 0.043 | 1.044 (0.714-1.526) | 0.826 |
| Lp-a | -0.001 | 0.999 (0.998-1.001) | 0.536 |
| HbA1c | 0.055 | 1.056 (0.998-1.118) | **0.057** |
| Urea | 0.027 | 1.028 (1.002-1.053) | **0.033** |
| Scr | 0.079 | 1.082 (0.905-1.293) | 0.386 |
| SAQ of Exertional capacity | -0.056 | 0.946 (0.930-0.962) | **＜0.001** |
| SAQ of Anginal stability | -0.019 | 0.981 (0.969-0.993) | **0.002** |
| SAQ of Anginal frequency | -0.027 | 0.973 (0.960-0.987) | **＜0.001** |
| SAQ of Disease perception | -0.012 | 0.988 (0.973-1.002) | **0.097** |
| SAQ of Treatment satisfaction | -0.063 | 0.939 (0.917-0.961) | **＜0.001** |

Bolded values represent *P* values < 0.2.

**Table S2. Univariate and multivariate Logistic regression on the absence of AR**

| Variables | Univariate | |  | Multiple | |
| --- | --- | --- | --- | --- | --- |
|  | OR (95% CI) | *P* values |  | OR (95% CI) | *P* values |
| Sex | 0.858 (0.490-1.503) | 0.592 |  |  |  |
| Age | 0.987 (0.964-1.010) | 0.272 |  |  |  |
| BMI | 0.869 (0.742-1.017) | 0.081 |  |  |  |
| Physical activity | 1.091 (0.640-1.858) | 0.749 |  |  |  |
| Smoking | 0.990 (0.574-1.707) | 0.971 |  |  |  |
| Alcohol consumption | 0.516 (0.295-0.903) | **0.020** |  |  |  |
| Hypertension | 0.974 (0.567-1.672) | 0.924 |  |  |  |
| Diabetes | 1.234 (0.715-2.131) | 0.450 |  |  |  |
| Hyperlipemia | 1.825 (1.068-3.119) | **0.028** |  | 1.858 (1.065-3.243) | **0.029** |
| Carotid atherosclerosis | 0.761 (0.482-1.353) | 0.352 |  |  |  |
| Stroke | 0.537 (0.189-1.523) | 0.242 |  |  |  |
| Renal insufficiency | 0.379 (0.051-2.836) | 0.344 |  |  |  |
| Antiplatelet | 4.771 (1.145-19.882) | **0.032** |  | 8.034 (1.506-42.858) | **0.015** |
| Antianginal | 0.626 (0.262-1.495) | 0.292 |  |  |  |
| Nitrate ester | 0.484 (0.215-1.090) | 0.080 |  |  |  |
| ACEI/ARB | 1.308 (0.770-2.222) | 0.321 |  |  |  |
| β-blockers | 1.257 (0.735-2.149) | 0.404 |  |  |  |
| CCB | 1.912 (1.115-3.278) | **0.018** |  | 1.923 (1.099-3.366) | **0.022** |
| Anticoagulant | 1.381 (0.599-3.184) | 0.449 |  |  |  |
| Lipid-lowering | 1.180 (0.490-2.838) | 0.712 |  |  |  |
| Gensini score | 0.997 (0.989-1.006) | 0.488 |  |  |  |
| Hcy | 1.019 (0.993-1.046) | 0.148 |  | 1.030 (1.002-1.058) | **0.035** |
| LDL-C | 1.275 (0.901-1.805) | 0.171 |  |  |  |
| Lp-a | 1.001 (0.999-1.002) | 0.392 |  |  |  |
| HbA1c | 1.068 (1.005-1.136) | **0.035** |  | 1.075 (1.013-1.142) | **0.018** |
| Urea | 0.998 (0.965-1.032) | 0.913 |  |  |  |
| Scr | 1.060 (0.879-1.278) | 0.541 |  |  |  |
| SAQ of Exertional capacity | 1.002 (0.986-1.019) | 0.793 |  |  |  |
| SAQ of Anginal stability | 0.998 (0.986-1.010) | 0.753 |  |  |  |
| SAQ of Anginal frequency | 1.005 (0.994-1.015) | 0.393 |  |  |  |
| SAQ of Disease perception | 1.014 (0.996-1.031) | 0.119 |  |  |  |
| SAQ of Treatment satisfaction | 1.012 (0.989-1.036) | 0.315 |  |  |  |
| Combined treatment | 0.484 (0.275-0.853) | **0.012** |  | 0.508 (0.283-0.910) | **0.023** |

Bolded values represent *P* values < 0.05.

**Table S3. Univariate and multivariate Logistic regression on the absence of angina attack**

| Variables | Univariate | |  | Multiple | |
| --- | --- | --- | --- | --- | --- |
|  | OR (95% CI) | *P* values |  | OR (95% CI) | *P* values |
| Sex | 0.564 (0.261-1.219) | 0.145 |  |  |  |
| Age | 1.001 (0.971-1.031) | 0.972 |  |  |  |
| BMI | 0.962 (0.789-1.173) | 0.702 |  |  |  |
| Physical activity | 0.566 (0.274-1.170) | 0.124 |  |  |  |
| Smoking | 1.640 (0.778-3.459) | 0.194 |  |  |  |
| Alcohol consumption | 0.681 (0.342-1.354) | 0.273 |  |  |  |
| Hypertension | 0.928 (0.467-1.847) | 0.832 |  |  |  |
| Diabetes | 1.600 (0.813-3.149) | 0.174 |  |  |  |
| Hyperlipemia | 1.365 (0.684-2.720) | 0.377 |  |  |  |
| Carotid atherosclerosis | 0.790 (0.382-1.634) | 0.790 |  |  |  |
| Stroke | 0.702 (0.210-2.344) | 0.565 |  |  |  |
| Renal insufficiency | 4.425 (1.576-12.421) | **0.005** |  | 3.903 (1.015-15.006) | **0.047** |
| Antiplatelet | 0.143 (0.071-0.286) | **＜0.001** |  | 0.265 (0.100-0.704) | **0.008** |
| Antianginal | 2.036 (0.928-4.468) | 0.076 |  |  |  |
| Nitrate ester | 0.928 (0.398-2.164) | 0.863 |  |  |  |
| ACEI/ARB | 1.456 (0.743-2.852) | 0.273 |  |  |  |
| β-blockers | 0.691 (0.352-1.357) | 0.282 |  |  |  |
| CCB | 1.036 (0.500-2.146) | 0.925 |  |  |  |
| Anticoagulant | 1.689 (0.632-4.514) | 0.296 |  |  |  |
| Lipid-lowering | 1.036 (0.357-3.012) | 0.948 |  |  |  |
| Gensini score | 1.037 (1.028-1.047) | **＜0.001** |  | 1.032 (1.021-1.043) | **＜0.001** |
| Hcy | 1.009 (0.973-1.047) | 0.624 |  |  |  |
| LDL-C | 0.923 (0.568-1.500) | 0.747 |  |  |  |
| Lp-a | 0.999 (0.997-1.001) | 0.525 |  |  |  |
| HbA1c | 1.072 (1.010-1.137) | **0.022** |  |  |  |
| Urea | 1.023 (0.992-1.054) | 0.143 |  |  |  |
| Scr | 1.097 (0.899-1.340) | 0.362 |  |  |  |
| SAQ of Exertional capacity | 0.935 (0.915-0.956) | **＜0.001** |  | 0.944 (0.915-0.973) | **＜0.001** |
| SAQ of Anginal stability | 0.979 (0.965-0.993) | **0.004** |  |  |  |
| SAQ of Anginal frequency | 0.969 (0.954-0.985) | **＜0.001** |  |  |  |
| SAQ of Disease perception | 0.984 (0.967-1.001) | 0.068 |  |  |  |
| SAQ of Treatment satisfaction | 0.926 (0.901-0.953) | **＜0.001** |  | 0.926 (0.892-0.962) | **＜0.001** |
| Combined treatment | 0.538 (0.265-1.094) | 0.087 |  | 0.230 (0.086-0.616) | **＜0.001** |

Bolded values represent *P* values < 0.05.

**Table S4. Logistic regression of univariate in PSM**

| Variables | Beta | OR (95% CI) | *P* values |
| --- | --- | --- | --- |
| Sex |  |  |  |
| Male | Reference |  |  |
| Female | -0.731 | 0.481 (0.237-0.980) | **0.044** |
| Age | 0.005 | 1.005 (0.976-1.034) | 0.753 |
| BMI | -0.036 | 0.964 (0.802-1.159) | 0.699 |
| Physical activity |  |  |  |
| Low | Reference |  |  |
| High | -0.963 | 0.382 (0.184-0.793) | **0.010** |
| Smoking |  |  |  |
| No | Reference |  |  |
| Yes | -0.127 | 0.880 (0.463-1.673) | 0.698 |
| Alcohol consumption |  |  |  |
| No | Reference |  |  |
| Yes | -0.272 | 0.762 (0.404-1.439) | 0.402 |
| Hypertension |  |  |  |
| No | Reference |  |  |
| Yes | -0.048 | 0.953 (0.498-1.821) | 0.884 |
| Diabetes |  |  |  |
| No | Reference |  |  |
| Yes | 0.514 | 1.672 (0.886-3.157) | 0.113 |
| Hyperlipemia |  |  |  |
| No | Reference |  |  |
| Yes | 0.265 | 1.303 (0.680-2.498) | 0.425 |
| Carotid atherosclerosis |  |  |  |
| No | Reference |  |  |
| Yes | -0.202 | 0.817 (0.408-1.637) | 0.569 |
| Stroke |  |  |  |
| No | Reference |  |  |
| Yes | 0.114 | 1.121 (0.423-2.974) | 0.818 |
| Renal insufficiency |  |  |  |
| No | Reference |  |  |
| Yes | 1.300 | 3.669 (1.151-11.695) | **0.028** |
| Combined treatment |  |  |  |
| No | Reference |  |  |
| Yes | -0.525 | 0.591 (0.310-1.129) | **0.111** |
| Antiplatelet |  |  |  |
| No | Reference |  |  |
| Yes | -2.011 | 0.134 (0.068-0.263) | **＜0.001** |
| Antianginal |  |  |  |
| No | Reference |  |  |
| Yes | 0.261 | 1.299 (0.579-2.912) | 0.526 |
| Nitrate ester |  |  |  |
| No | Reference |  |  |
| Yes | -0.164 | 0.848 (0.382-1.886) | 0.687 |
| ACEI/ARB |  |  |  |
| No | Reference |  |  |
| Yes | 0.577 | 1.780 (0.942-3.363) | **0.076** |
| β-blockers |  |  |  |
| No | Reference |  |  |
| Yes | -0.263 | 0.769 (0.409-1.447) | 0.415 |
| CCB |  |  |  |
| No | Reference |  |  |
| Yes | 0.024 | 1.024 (0.510-2.056) | 0.947 |
| Anticoagulant |  |  |  |
| No | Reference |  |  |
| Yes | 0.223 | 1.250 (0.424-3.683) | 0.686 |
| Lipid-lowering |  |  |  |
| No | Reference |  |  |
| Yes | 0.318 | 1.374 (0.409-4.616) | 0.607 |
| Gensini score | 0.040 | 1.041 (1.031-1.051) | **＜0.001** |
| Hcy | 0.015 | 1.015 (0.976-1.055) | 0.450 |
| LDL-C | -0.250 | 0.799 (0.486-1.249) | 0.300 |
| Lp-a | -0.001 | 0.999 (0.996-1.001) | 0.217 |
| HbA1c | 0.002 | 1.002 (0.870-1.153) | 0.981 |
| Urea | 0.022 | 1.022 (0.990-1.055) | **0.177** |
| Scr | -0.030 | 0.971 (0.673-1.400) | 0.874 |
| SAQ of Exertional capacity | -0.061 | 0.941 (0.921-0.960) | **＜0.001** |
| SAQ of Anginal stability | -0.020 | 0.980 (0.967-0.994) | **0.004** |
| SAQ of Anginal frequency | -0.029 | 0.972 (0.957-0.987) | **＜0.001** |
| SAQ of Disease perception | -0.016 | 0.984 (0.968-1.000) | **0.054** |
| SAQ of Treatment satisfaction | -0.074 | 0.929 (0.904-0.954) | **＜0.001** |

Bolded values represent *P* values < 0.2.
