# Supplementary figures and images for: Integrated Chinese and Western medicine for stable angina pectoris of coronary heart disease: a real-world study including 690 patients
Source: Front Cardiovasc Med. 2023 May 19;10:1194082. doi: 10.3389/fcvm.2023.1194082 (PMC10235782; doi:10.3389/fcvm.2023.1194082)

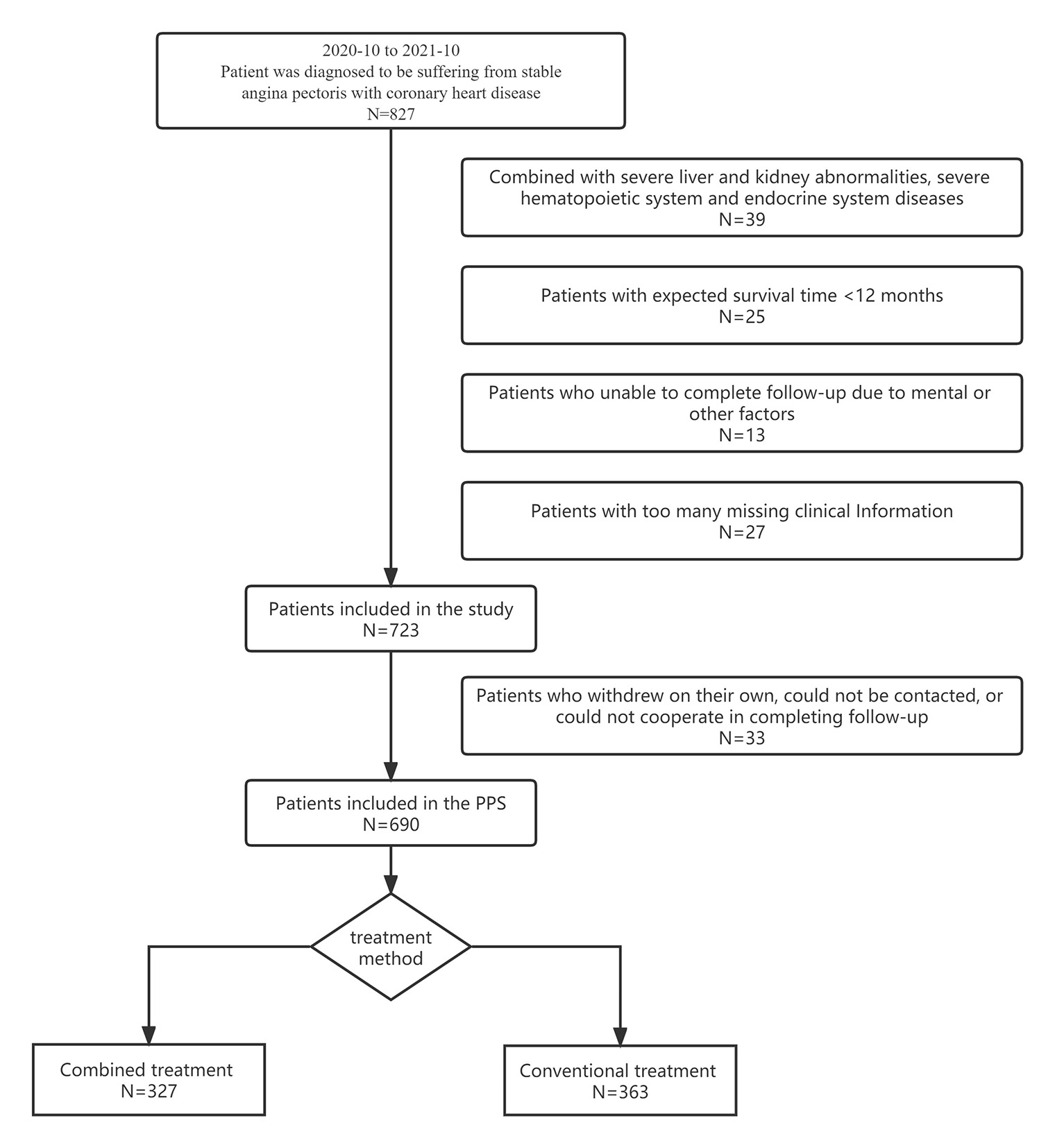

Supplement: Supplementary file 3 [file Image1.jpeg]
